# Measuring the relationships between various urban green spaces and local climate zones

**DOI:** 10.1038/s41598-023-36850-6

**Published:** 2023-06-16

**Authors:** Vlaďka Kirschner, Karel Macků, David Moravec, Jan Maňas

**Affiliations:** 1 Department of Landscape and Urban Planning, Faculty of Environmental Sciences, Czech University of Life Sciences Prague, 16500 Prague, Czech Republic; 2 Department of Geoinformatics, Palacký University in Olomouc, Olomouc, Czech Republic; 3 Department of Spatial Sciences, Faculty of Environmental Sciences, Czech University of Life Sciences Prague, 16500 Prague, Czech Republic

**Keywords:** Climate sciences, Environmental sciences

## Abstract

Urban green spaces (UGS) improve living conditions in cities by mitigating the Urban Heat Island effect. While the cooling effect of UGS seems unequivocal, the relationship between the types of UGS and types of residential areas has not yet been well explored. In this study, we systematically analysed the cooling effect of 71 UGS in Prague, a central European city, on residential areas within 400 m of the UGS. The UGS are classified according to their spatial characteristics (size, shape, and tree density), and the residential areas according to three Local Climate Zones (LCZ 2, 5, 6) typical for European cities. The cooling effect is evaluated using a regression model of the Land Surface Temperature (LST) in residential zones according to the LCZ type and distance from the various UGS. The results show that compact UGS of 10–25 ha with dense trees have the most pronounced cooling effect. This type of UGS was associated with a mean decrease in LST within 400 m of 2.3 °C compared to the least effective UGS type (long with sparse trees) across LCZs. The results of the presented study can be applied in urban planning and urban design to improve microclimates in cities.

## Introduction

Planning policies across the globe aim to create sustainable, resilient, and healthy places to live. With rising populations in cities and the increasing frequency of extreme heat events^1^, these aims are gaining in importance. Urban Green Spaces (UGS) have been broadly recognised as an effective nature-based solution in urban heat mitigation, cooling the temperature and improving the urban microclimate in their surroundings, especially in drier climate conditions^2–4^. The cooling effect of UGS might be further enhanced by proposing UGS with appropriate characteristics^5^. The presence of accessible UGS brings further positive effects, especially to residents with reduced mobility, such as children, older people, or people with cardiovascular diseases, whose exposure to high temperatures is associated with greater health risks^6^. In short, a balanced distribution of appropriately designed UGS throughout cities can work both as a prevention of various diseases^7^ and as an effective mitigation measure against the formation of Urban Heat Islands (UHI).

The cooling effect of UGS was recognised to be substantial, especially in dry European conditions^2,3^. Consequently, the characteristics of UGS were studied with the maximisation of the UGS’ mitigation effect in mind. Three characteristics of the UGS’ cooling effect are mentioned particularly often as the most influential: size, shape, and tree density. Starting with the latter, most studies^8–11^ agree that trees provide higher cooling efficiency than grass. For instance, a park near Lisbon (Portugal) with a prevalence of trees was found to influence temperature significantly within 60 m, while a park with a prevalence of grass only influenced the LST within 10 m^9^. The maximum cooling distance observed for forests and urban parks in Leipzig (Germany) was 469 m and 391 m, respectively^11^.

UGS with a regular and compact shape were found to be more effective at cooling than those with irregular and elongated shapes^10,12,13^. Larger UGS are more effective at cooling than small ones^10,14^, but many studies suggest that there are a lower and upper threshold of efficiency. The value of the thresholds, however, varies among cities. For instance, a negligible effect was found for UGS smaller than 5.6 ha in Leipzig^11^, 2–3 ha in London^13^, and 2 ha in Shanghai^12^. In contrast, in Almada (Portugal), UGS of even 0.49 ha with a high tree density alleviated the temperature by at least 1 °C^9^. Based on an extensive literature review, Aram et al. (2019) established the lower threshold on UGS to be 10 ha, with a 1–2 °C effect on surrounding areas within 350 m. The upper threshold size of 40 ha was measured in Shanghai^15^.

Overall, the cooling effect of UGS and the role of their size, shape, and tree density seem unequivocal. Nevertheless, the relationship between various types of UGS and the surrounding urban structures within the context of walkable distance has not been sufficiently explored. Many studies of UGS have been performed without considering the surrounding context^9,10,13^, even though the effect of city structure (also called city morphology) is well known^16^. Only a few studies measured the UGS’ effect in the context of the city structure. For instance, a Chinese study^8^ described the city structure using various indices considering the size of the area, population density, and night light intensity. Although incorporating the typomorphological classification of the city structure into the Local Climate Zone (LCZ) framework^17^ is well established and is used to capture the variation that characterises neighbourhood microclimates (and although a global LCZ map has been created by Demuzere et al.^18^), the LCZ framework has been used only rarely for studying the effects of UGS. In addition, more than 60% of LCZ studies were conducted in China, followed by the United States. In terms of European countries, most studies on this topic come from Germany, but even so, only 17% of articles concerning LCZs in the last ten years have been performed in that country, which indicates the lack of research on this topic in the European context^19^. To the best of our knowledge, only UGS within open midrise (LCZ 5) and compact midrise (LCZ 2) city structures have been analysed in Europe (in Germany)^11^.

In this study, following the recommendation of Rakoto^20^, we want to explore the relationship between various UGS (characterised by size, shape, and tree density) and temperature in various residential areas (characterised by LCZ) in the city of Prague, located in central Europe. We intend to measure the influence of UGS on various LCZ units within a perimeter of 400 m, a distance considered a threshold of walkability across the globe^21^, corresponding to approximately 5–10 min of walking^22^. The rationale behind the research is to contribute to designing and planning UGS that effectively mitigate UHI within a walkable city with a balanced distribution of UGS.

## Methods

The article focuses primarily on the relationship between the types of UGS and LCZs on a grid scale. First, we needed to define the grid scale of the study; we based it on a grid of LST as a prerequisite for the estimation of the UHI effect. Then, we defined and categorised 71 UGS and selected units within 400 m of these UGS, in which LST was measured. Grid squares of residential areas within 400 m of UGS meeting the inclusion and exclusion criteria were selected for the study and classified based on three types of LCZs typical of the city of Prague. Each such 90 × 90 m grid square then constituted an individual unit for further evaluation. Finally, analyses were performed in order to explore the influence of various types of UGS on LST in three types of LCZ units. The methods are described in more detail below.

### Definition of LCZ units

The study area comprises the city of Prague, the capital of the Czech Republic (50°5′N, 14°25′E). The Czech Republic has a temperate climate, being situated on the border of the western oceanic and the eastern continental climates, with the typical pattern of four seasons. The Land Surface Temperature (LST) within Prague, a prerequisite for quantifying the surface UHI effect, was calculated from an image acquired at 11:50 a.m. local time, on June 26, 2019, under clear atmospheric conditions when the air temperature in the centre of Prague (Karlov weather station) was 31.2 °C and the wind speed was 1.8 m s^−1^. The image was taken by satellite Landsat-8 TIRS Band 10, with a ground resolution of 100 m resampled to 90 m. The 90 × 90 m raster corresponds with the spatial resolution at which the cooling effect of greenery was reported to be most pronounced in the United States and China^23,24^. The LST was obtained from the top of the atmosphere radiance in the original satellite using a method proposed by Barsi et al.^25,26^. The method contained the following: firstly, conversion from digital numbers to the radiance values; secondly, atmospheric correction of radiance values using parameters obtained from the Atmospheric Correction Parameter Calculator (available online: https://atmcorr.gsfc.nasa.gov/); lastly, the radiance values emissivity normalisation according to a procedure proposed by Van De Griend & Owe^27^. Finally, the corrected radiance values were converted to °C. The data are in Krovak East North system (SJTSK).

**Figure 1:**
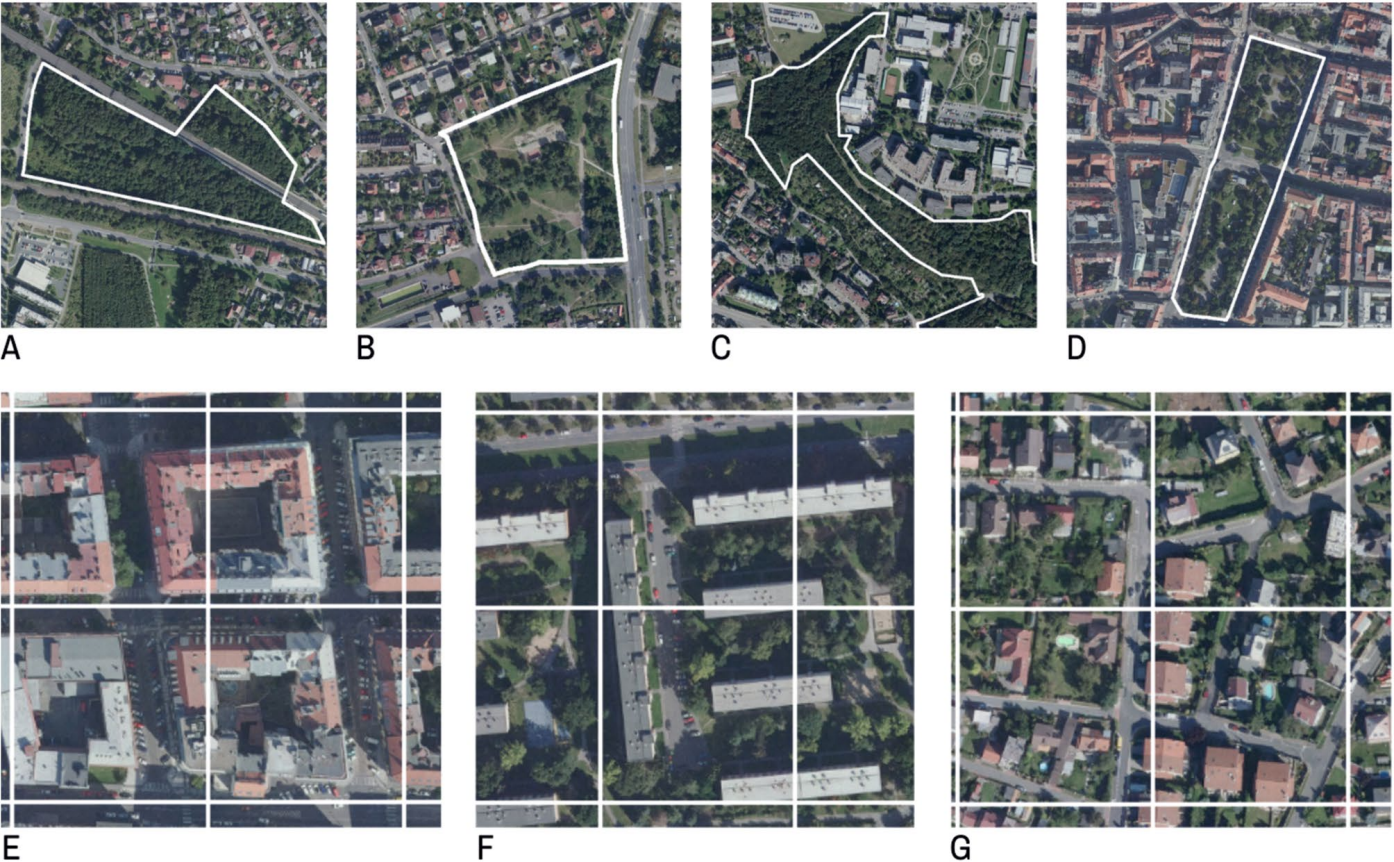
Representative examples of UGS: Compact Dense (**A**), Compact Sparse (**B**), Long Dense (**C**), Long Sparse (**D**). Representative examples of LCZ: LCZ 2 (**E**), LCZ 5 (**F**), and LCZ 6 (**G**) displayed on a 90 × 90 m raster. Created in ArcGIS PRO 3.1^29^ based on WMS-orthophoto (publicly available at © ČÚZK, Czech Office for Surveying, Mapping and Cadaster).

Firstly, 71 UGS were chosen within Prague's residential areas and classified according to their size, shape, and tree density, based on the results of previous research^5,8,10,14,24,28^. Most UGS chosen were of a compact shape (Fig. 1a, b); these were further divided according to their size into Small (2–7 ha, 18 UGS), Medium (10–25 ha, 16 UGS), Large (50–95 ha, 14 UGS), and Extra large (120–490 ha, 5 UGS). If the length of a UGS greatly exceeded its width, the UGS was designated as Long (2–50 ha, 18 parks; Fig. 1c, d). UGS with less than 40% trees were classified as Sparse (30 parks; Fig. 1b, d), and UGS with a prevalence of trees (more than 60%) were categorised as Dense (41 parks; Fig. 1a, c); all Extra large UGS were UGS with dense trees. We excluded UGS covered only with grass from the study, assuming, in accordance with previous studies, their negligible cooling effect^3,8,20^.

Secondly, residential study areas in the vicinity of UGS were selected. We based this selection on the raster of LCZs globally defined and accessible by Demuzere^18^ using the WGS84 system. The raster is at 100 m resolution, which roughly corresponds to the 90 m resolution of the LST map. To make the raster comparable with our grid, it was transformed into SJTSK system and adjusted to a 90 × 90 grid. Three zones were the most common within the residential areas of Prague: compact midrise (LCZ 2), open midrise (LCZ 5), and open low-rise (LCZ 6), which is consistent with the dominant zones in European cities, i.e., cities belonging to the cluster 3 according to^30^. Comparing the LCZ raster with the orthophoto map of Prague, several differences in city structure emerged, stemming from the fact that the LCZ definition^17^ does not reflect detailed morphological parameters within neighbourhoods^31^. In order to avoid possible confounding effects arising from this fact, we narrowed down the specification of the three zones. Only 4 to 6-storey block structures were considered LCZ 2 (Fig. 1e), only 6 to 9-storey housing estates typical for the communist era were considered LCZ 5 (Fig. 1f), and only detached and semi-detached family houses were considered LCZ 6 (Fig. 1g). Despite this narrower specification, in this study we still use the original designations of LCZ 2, 5, and 6.

In this study, we aim to measure, specifically, the influence of the UGS on the surface UHI effect. Therefore, we selected only units on which there would be a minimal possible influence of other factors. Firstly, we did not select units located centrally among multiple UGS to avoid the multiple effects of greenery^11,14^. Secondly, we excluded areas within approximately 200 m of the river and 100 m of lakes from the selection, considering the water-cooling effect^3,32^. Thirdly, taking into account spatial autocorrelation issues^33^, we focused only on areas surrounded by a single type of LCZ. Other significant city morphology factors, such as paved and built-up areas, are already considered within the characteristics of individual LCZs.

**Figure 2:**
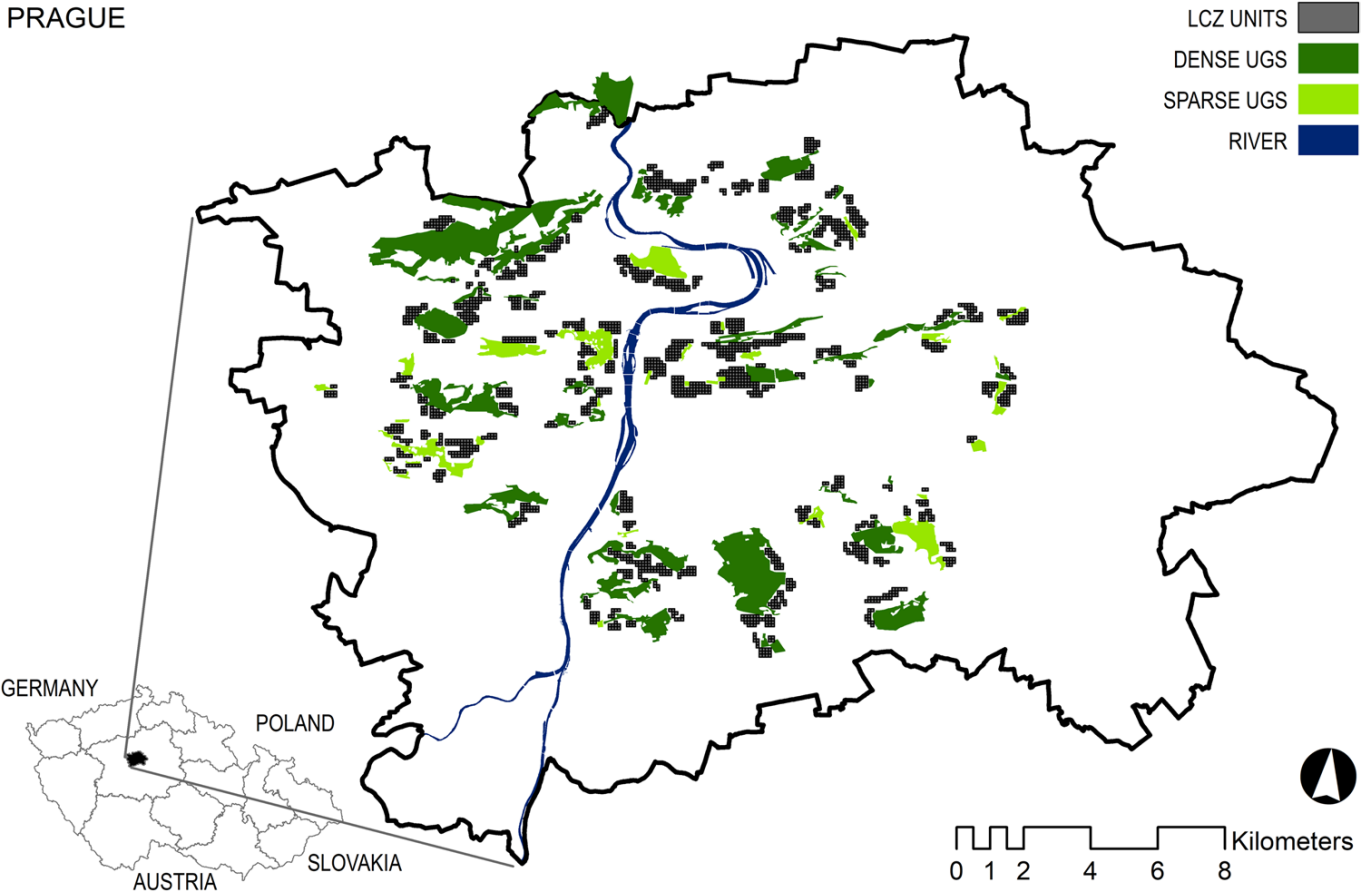
The map of LCZ units within 400 m of UGS (UGS are displayed with tree density category).

For further analysis, the built-up areas of LCZs were split in the GIS environment into a regular 90 × 90 m grid according to the geometric structure of the LST data. Each newly created cell was assigned an LST value. Subsequently, each cell was associated with the attributes of its nearest UGS—its type, size, and distance from the cell’s centroid to the nearest UGS using the NNJoin tool in the QGIS environment. Finally, only cells that were within a buffer of 400 m from the UGS boundary (i.e., the distance between the centre of the LCZ unit and the boundary) were considered for further analysis. The LCZ units included in the analysis and their position relative to the UGS are presented in Fig. 2. Thus, processed data were used as the input for the statistical analysis.

### Measuring the relationship between UGS and LST in LCZ units

Firstly, the difference between the mean value of LST in three different LCZ categories (groups) was analysed. Both visual analysis methods (boxplot) and statistical tests were applied. Usually, analysis of variance—ANOVA^34^—is used to test any difference between groups. However, one of the assumptions of ANOVA is the normality of data (LST in our case) distribution in each group. Therefore, the normality was tested with the Kolmogorov–Smirnov test^35^; it did not prove normal distribution of the LST within the groups. Following the test result, a non-parametric alternative of ANOVA, i.e., the Kruskal–Wallis test^36^, was used. The test confirmed the difference in LST between the groups. Secondly, the statistically significant difference in means values of LST between the pair groups was identified by running the Nemenyi post-hoc test^37^.

To model the effect of UGS on the temperature of the built-up area, a descriptive linear model with quantitative and qualitative predictors was constructed:

$$Y= {\beta }_{0}+{\beta }_{1}{X}_{1}+{\beta }_{2}{X}_{2}+\dots +{\beta }_{p}{X}_{p}+\varepsilon $$

as proposed by James et al.^38^, where $Y$ is the dependent variable, ${\beta }_{0}$ is the intercept, ${X}_{1}\dots {X}_{p}$ represent predictor matrices, ${\beta }_{1}\dots {\beta }_{p}$ are their coefficients, and $\varepsilon$ is the vector of random errors. The quantitative predictor distance expresses the distance of the observed development unit from the nearest UGS; the LCZ category, the UGS’s size, and the UGS’s type served as qualitative predictors. For this reason, the approach of dummy variables has been implemented using the variables of UGS size (with levels of Small, Medium, Long, Large, or Extra-large), park type (Dense or Sparse), and LCZ (LZC 2, LZC 5, or LZC 6). The levels Extra-large, Sparse and LZC 2 were considered to be the baseline for the UGS size, type, and LCZ, respectively. In the interpretation of qualitative predictors, the coefficients of particular levels are compared to the baseline to allow for the evaluation of the differences among levels of individual qualitative predictors.

## Results

### Comparing LST in LCZ and types of UGS

**Figure 3:**
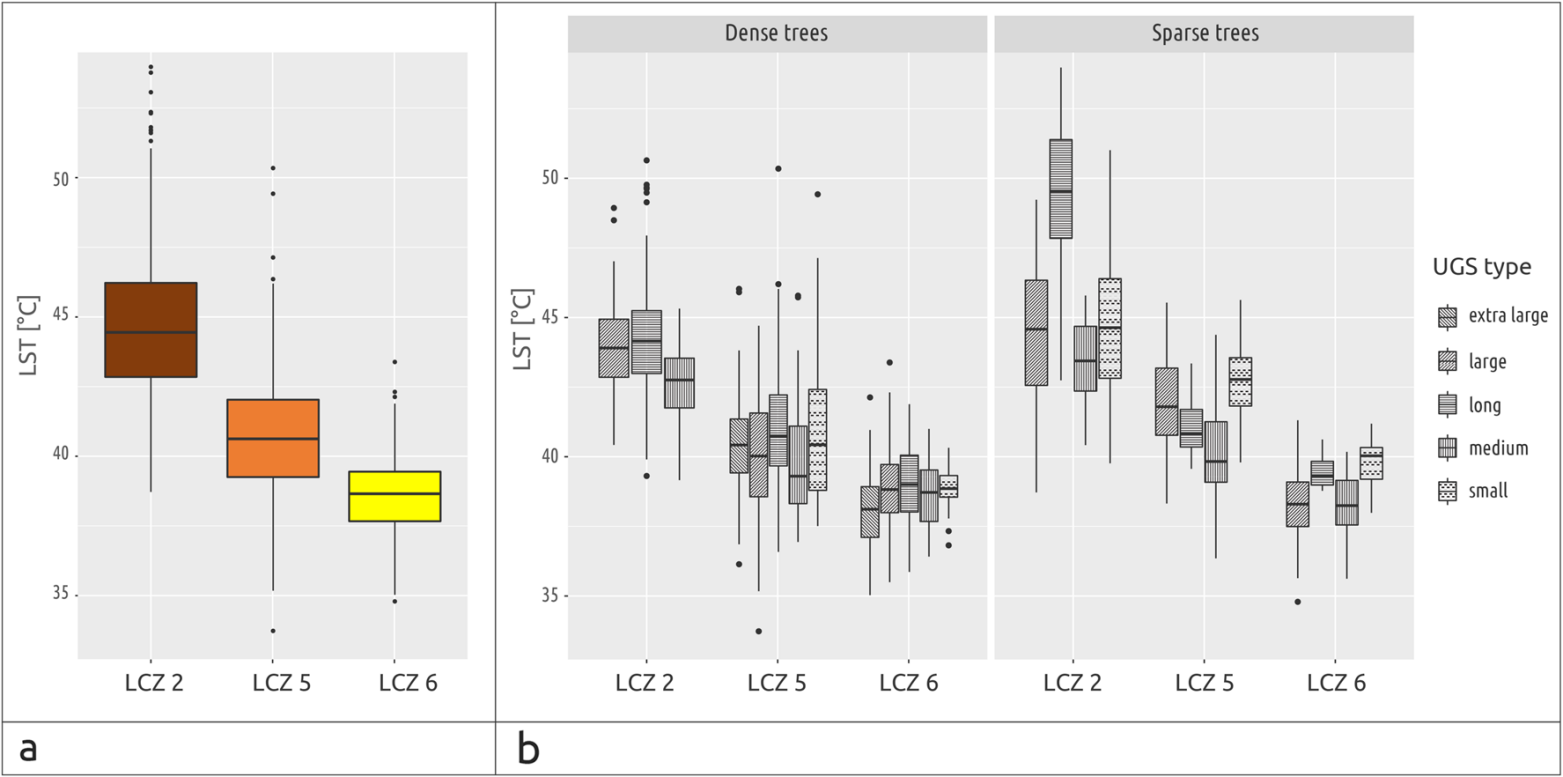
Comparison between LST in 3 categories of LCZs in general (**a**), and according to tree density and UGS type (**b**).

The LST was measured in 2,036 LCZ units located within 400 m of 71 UGS. The units were classified into three categories (LCZ 2, 5, and 6) containing 487, 848, and 701 grids, respectively. The differences in the mean value of the LST in 3 categories of LCZ were analysed by the Kruskal–Wallis test. The null hypothesis stating that groups’ medians are equal has been rejected at the significance level $\alpha =0.01$. The subsequent post-hoc Nemenyi test indicated a statistically significant difference between all pairs of groups. The LST in 3 LCZs are presented as a boxplot in Fig. 3a. The highest LST was observed in LCZ 2, followed by LCZ 5 and LCZ 6, with average LSTs of 44.71 °C (± 2.66), 40.69 °C (± 2.04), and 38.57 °C (± 1.27), respectively.

More complex results classified according to tree density and UGS type are shown in Fig. 3b. Although the effect of the LCZ can still be well recognised, we can see also differences based on the UGS type. Exceptionally high LSTs can be observed in LCZ 2 in the vicinity of Long Sparse UGS. A higher LST can also be noted around the Small Sparse UGS in LCZ 5 and 6. A more detailed analysis is presented below.

### Associations between LST, UGS, and LCZ

A regression model including the UGS type, UGS tree density, LCZ unit category, and its distance from the UGS was constructed to predict LST in LCZ units. The LCZ category, type of UGS, and tree density in the UGS were used as categorical predictor variables. LCZ 2, Extra large UGS, and Dense UGS were considered the baseline values, to which the other categories were compared. The model uses dummy coding for the calculation of the average estimated LST for each combination of predictors. The regression model is presented in Table 1, and the dummy coding in online Appendix 1. All levels of the considered qualitative variables proved to be statistically significant at the significance level of $\alpha =0.01$, except for the Large UGS, the effect of which was comparable with that of Extra large UGS.

**Table 1:** Regression model.

Predictor	Coefficient (± SD)	t statistics	p value
Intercept	42.726 ± .177	241.77	< 2e−16
distance	0.005 ± .000	12.50	< 2e−16
LCZ 6	− 5.352 ± .123	− 43.67	< 2e−16
LCZ 5	− 3.457 ± .113	− 30.53	< 2e−16
Large UGS	− **0.007** ± .141	− **0.05**	**0.9581**
Long UGS	0.897 ± .152	5.91	0.0000
Medium UGS	− 0.552 ± .161	− 3.43	0.0006
Small UGS	0.495 ± .176	2.82	0.0049
Sparse UGS	0.844 ± .099	8.56	<< 0.001

The only statistically insignificant variables are highlighted in bold.

This model explained 63% of the data variability. Interpreting the baseline variables, the model estimated that the LST in LCZ 2 would increase by 0.5 °C with every 100 m from the Extra-large Dense UGS. The average LST in LCZ 5 was 3.46 °C lower than in LCZ 2, and the LST in LCZ 6 was 1.90 °C lower than in LCZ 5. The differences in LST associated with the remaining categorical predictors are not as large as in the case of LCZ predictors. The LST in units adjacent to Sparse UGS is, on average, 0.84 °C higher than in those in the vicinity of Dense UGS. The LST around Large UGS is not significantly different from that around Extra-large UGS (p > 0.05). Of the other types of UGS, i.e., Long, Medium, and Small UGS, the LST in their vicinity was affected by + 0.90, − 0.55, and + 0.50 °C compared to Extra large UGS, respectively.

**Figure 4:**
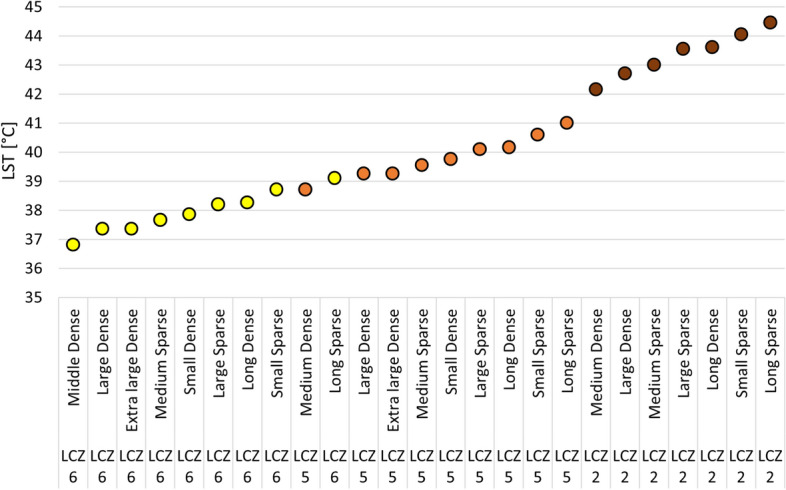
The graph of the estimated LST. Colours: yellow = LCZ 6, orange = LCZ 5, brown = LCZ 2.

Figure 4 depicts the individual unit types in the order of increasing LST. It is obvious that LST generally increases from LCZ 6 to LCZ 5 and again to LCZ 2, regardless of the tree density and UGS type. Within each LCZ, the highest LSTs were observed in the vicinity of Long Sparse UGS. LCZ 6 units in the vicinity of this type of UGS are even warmer than LCZ 5 areas adjacent to the Medium Dense UGS. The second highest LST is observed in the vicinity of Small Sparse UGS, followed by Long Dense, Large-Sparse UGS in all LCZ. The further order slightly varies between LCZ. The LSTs in the vicinity of Extra-large Dense and Large Dense UGS are not significantly mutually different (note that there were no Extra large UGS in the vicinity of LCZ 2). In short, regardless of the tree density and the LCZ, the Medium UGS always show the highest cooling effect, followed in the same (descending) order: Large, Extra large, Small, and Long UGS.

## Discussion

The presented study evaluates the main relationships between the LST, urban structures (represented by 3 LCZs with detailed structure specification), and UGS characteristics (defined size, tree density, and shape of the UGS) in units situated within 400 m of 71 UGS in Prague. The following discussion aims to contribute towards the application of our results in urban design and planning, while at the same time considering the limitations of the study and outlining further research directions. Overall, our results confirm that the LST is affected both by the UGS’ properties and the urban characteristics of the respective location^15^, with the urban characteristics (represented in this study by LCZ) being more influential than those of green spaces.

The block structures in LCZ 2 were identified as the warmest of the three zones measured, which is in accordance with findings from other European cities (Prague, Brno, Novi Sad—Serbia, and Szeged—Hungary) and from the semi-humid, warm, and temperate continental monsoon climate of China^39–41^. Considering that the LCZ 2 remains the warmest zone even in the vicinity of UGS of various sizes and densities, the LCZ 2 appears to be the zone with the most urgent need for UHI mitigation. Integrating appropriate UGS in the area may reduce the LST by up to 2.3 °C. However, even this reduction appears to be insufficient, calling for a more significant LST reduction within this zone. Therefore, we propose combining UGS formation with other mitigation measures, including integrating other types of greenery (e.g., green roofs, greenery on façades) or water bodies, permeable surfaces, application of building materials with low albedo, or considering the shadowing effect of buildings with different heights.

In line with previous research^39,40^ family houses in LCZ 6 are slightly cooler than housing estates in LCZ 5. While the differences in LST between LCZ 5 and 6 are relatively small (2.12 °C ± 1.66) compared to the differences between LCZ 2 and 5 (4.02 °C ± 2.35), the difference in residential density between the two zones is massive. In Prague, the population density in block structures (LCZ 2) and housing estates (LCZ 5) is approximately 170–190 inhabitants per hectare, which drops to approximately 30–40 inhabitants per hectare in the zones with family houses (LCZ 6)^42^. Therefore, considering the efficiency of land use combined with temperature comfort, the housing estate in LCZ 5 appears to be a suitable compromise. As the sealed area (including the built-up area) in LCZ 5 is far smaller than in LCZ 2, integrating relevant UGS in the existing structure will be easier than in LCZ 2. The formation of relevant UGS (medium-sized with dense trees) could reduce the LST to the level of LCZ 6 (see Fig. 4) in the summer in Prague, i.e., the LCZ with the lowest LST from all zones (apart from "sparsely built"—LCZ 9—which is, however, not considered a city-forming structure)^39^.

Based on our results, we cannot fully confirm that larger UGS have a greater cooling effect than smaller ones, as reported in previous studies^15,20,33^. The greatest cooling effect was observed for compact UGS of 10–25 ha (Medium UGS). In our study, as the size of an UGS increased, the effect even decreased; Large UGS (50–95 ha) had a lower cooling effect than Medium ones. The smallest reduction in LST was found in the vicinity of Small UGS (2–7 ha); assuming a linear change in temperature with distance, the LST in LCZ 2 grows by 0.3 °C per 100 m from the boundary of Small Dense UGS. A slightly higher cooling effect was found in London^43^, where the 3–5 ha UGS cooled the area within 100–150 m by 0.7 °C. However, the cooling effect was measured at night and did not consider the surrounding structure. Overall, we can conclude that in residential areas of LCZ 2, 5, and 6, the UGS' cooling effect increases with their size up to the threshold of somewhere between 25 and 50 ha, which is in line with the threshold of 40 ha reported in Shanghai^15^.

LCZ units within 400 m of the Long Sparse UGS were consistently the warmest in each LCZ, with Long Dense performing slightly better. The elongated shape was also found to be less effective in cooling in London^13^. The most likely explanation lies in the fact that their narrow width does not support the formation of an interior microclimate^10^. Nevertheless, the Long UGS still have an incommutable place within the urban structure; they connect urban spaces, improving accessibility within the city. Some studies^28,33^ also reported that the UGS’ cooling effect increases if they are mutually interconnected, which may be considered an important function of Long UGS. To conclude, Long UGS should be proposed with dense trees, creating green connections between different spaces.

In line with other studies, we confirm that the cooling effect increases with tree density^9–11,23,28^. The results showed a very clear order of the type of UGS (when considering Dense and Sparse separately). The cooling efficiency of UGS is (in descending order): Medium, Large + Extra large, Small, and Long, irrespective of the city structure. When combining the tree density with the UGS type, the order is somewhat changed. For example, Small Dense UGS are more efficient than Large Sparse UGS, and Long Dense UGS are more efficient than Small Sparse UGS. In these cases, the tree density is more important than the size and shape of the UGS. However, the size is crucial when comparing the Medium and Small UGS: Medium Sparse UGS are more efficient at cooling the surroundings than Small Dense UGS. Nevertheless, Medium and Large Dense UGS are always the coolest, and Long and Small Sparse UGS are always the warmest. All these relationships are relevant for all LCZs measured. Similar conclusions were drawn in Almada (Portugal), where increased tree density in Small UGS reduced the temperature by 1–3 °C^9^ and in London, where Small UGS reduced the temperature within 440 m by 1.1 °C^13^. Our results are also in line with the conclusions by Aram et al.^44^, who found a clear cooling effect on the areas within 350 m of UGS larger than 10 ha. Overall, our results show that the effects of the tree density, size, and shape of UGS play a role in LST cooling, and all these factors must be considered together with the surrounding urban structure for effective UHI mitigation.

Our research did not aim to determine an exact threshold of the best-performing UGS size for cooling the surroundings. The reasons were twofold: (1) it was necessary to categorise the UGS to obtain a sufficient number of UGS for research and (2) in our study, we do not provide a continuous range of UGS sizes. Further research might refine the appropriate ranges; however, from the perspective of practical spatial planning working with limited areas designated for creating UGS, the finding that the lower threshold for efficient cooling is 10 ha is probably sufficient. Further research should concentrate rather on increasing the efficiency of Small, Medium, and Large Dense UGS in LCZ 2 and 5, while both vegetation structural and functional aspects could be included in such research—including the capacity of vegetation to withstand increasing heat associated with climate change. It would also be beneficial to repeat this study across other Köppen climate classification types as well as across different local climate zones.

Our research only considered areas within 400 m of UGS, which makes the results applicable in planning and designing residential development on a local scale. To make the results applicable to larger-scale planning (e.g. in urban plans), a greater study area would be needed. Similarly, apart from the size, tree density, and shape, the spatial configuration of UGS (i.e., their location within the city) also affects the LST^28,33^ and, hence, should be further studied in the larger scale context, especially in European conditions.

## Conclusion

The UHI effect requires cities to pay greater attention to mitigation measures when planning and designing residential areas. This study focuses on UGS as an effective measure in cooling the urban environment. We investigated the relationship between LST in different LCZs in the vicinity of UGS in Prague (central Europe). The results contribute to urban design and planning as follows:

- The LST within 400 m of UGS depends more on the type of LCZ than on the type of UGS. LCZ 2 is the hottest LCZ, followed by LCZ 5 and LCZ 6, with a total mean difference of 6.1 °C. Within each LCZ, the LST differs by 2.3 °C. This value, therefore, indicates the LST improvement achievable by proposing the appropriate size, shape, and tree density of UGS.
- The medium size of compact UGS (i.e., 10–25 ha) is the most efficient at cooling the surrounding environment. The cooling effect decreases when increasing the size of UGS to more than 50 ha. The cooling effect can be increased by increasing tree density to more than 60%.
- Long UGS, followed by Small compact UGS (2–7 ha) covered prevalently by grass, are the least effective at cooling their neighbourhood. Long UGS should be preferably designed as dense greenery and connected to other greenery. By increasing the tree density to more than 60%, the Small UGS becomes more efficient at cooling than the Large UGS covered prevalently by grass, and the Long UGS becomes more efficient than the Small UGS covered prevalently by grass.
- The aforementioned principles should be integrated with priority into LCZ 2 and LCZ 5. In LCZ 2, however, the defined principles must be combined with other mitigation measures in order to achieve adequate LST reduction.

This study improves our understanding of how UGS influence the surroundings. Urban planners and architects can refer to the conclusions to improve urban microclimates and thus create a comfortable living environment.

## Supplementary Information

Supplementary Information.

## Data Availability

All data generated or analysed during this study are included in this published article.
