# Supplementary material for: Measuring the relationships between various urban green spaces and local climate zones
Source: Sci Rep. 2023 Jun 16;13:9799. doi: 10.1038/s41598-023-36850-6 (PMC10275979; doi:10.1038/s41598-023-36850-6)
Supplement: Supplementary file 1 — Supplementary Information. [file 41598_2023_36850_MOESM1_ESM.pdf]

	sparse trees
**dense trees**	0
sparse trees	1

	LCZ 6	LCZ 5
**LCZ 2**	0	0
LCZ 6	1	0
LCZ 5	0	1

	large	long	medium	small
**extra large**	0	0	0	0
large	1	0	0	0
long	0	1	0	0
medium	0	0	1	0
small	0	0	0	1
